# Late Clinical Presentation Outweighs Viral Characteristics in Determining Immune Depletion at HIV Diagnosis: A Five-Year Retrospective Study

**DOI:** 10.3390/v18090986

**Published:** 2026-09-08

**Authors:** Silvere D. Zaongo, Mei Han, Yaokai Chen

**Affiliations:** 1 Clinical Research Center, Chongqing Public Health Medical Center, 109 Baoyu Road, Geleshan Town, Shapingba District, Chongqing 400036, China; zsildieu@yahoo.fr (S.D.Z.); harmonyhan@126.com (M.H.); 2 Department of Infectious Diseases, Chongqing Public Health Medical Center, 109 Baoyu Road, Geleshan Town, Shapingba District, Chongqing 400036, China

**Keywords:** HIV-1 subtypes, late presentation, immune depletion, baseline data

## Abstract

Background: Late presentation and poor immunovirological outcomes persist among people living with HIV (PLWH). The differential impact of HIV-1 subtypes on baseline disease presentation in antiretroviral therapy (ART)-naïve individuals remains incompletely characterized, particularly in regions dominated by unique recombinant forms. Methods: We conducted a retrospective cross-sectional study involving ART-naïve newly diagnosed PLWH at our hospital from January 2020 to October 2025. Immunological (CD4+, CD8+ T-cell counts, and CD4+/CD8+ ratios), biochemical [albumin (ALB), alanine aminotransferase (ALT), aspartate aminotransferase (AST)], virological (HIV-1 subtype, genotypic drug resistance), and clinical (admission type, age) parameters were analyzed. Multivariate linear regression identified factors independently associated with CD4+ T-cell count, CD4+/CD8+ ratio, and ALB level. Results: The study population comprised 762 ART-naïve newly diagnosed PLWH who were predominantly male (87.5%), older (median age 59), and presented with advanced immunosuppression (median CD4+ count 100 cells/µL). CRF07_BC was the dominant subtype (57.4%). Significant subtype-specific differences were observed: CRF01_AE infection was associated with the lowest CD4+ counts (median 54 cells/µL, *p* = 0.0004) and CD4+/CD8+ ratios (*p* = 0.0005). Inpatient status, indicating more severe presentation, was independently associated with significantly lower CD4+ counts (β = −36.91, *p* < 0.0001) and ALB levels (β = −7.358, *p* < 0.0001). Contrary to typical immune aging, patients aged ≥50 years had higher CD4+ counts at diagnosis (105.5 vs. 74 cells/µL, *p* = 0.0039). Genotypic drug resistance (22.2% prevalence) showed no independent association with baseline immunological status. Conclusions: Our findings reveal a distinct profile of late HIV presentation in a contemporary Chinese cohort, characterized by advanced age and the predominance of CRF07_BC. The independent association of inpatient status, rather than HIV-1 subtype or drug resistance, with severe immunosuppression and hypoalbuminemia highlights clinical presentation as the paramount indicator of baseline disease severity. The unexpected immunological advantage in older adults warrants further investigation.

## 1. Introduction

Despite major advances in antiretroviral therapy (ART), late presentation to HIV care, defined as a CD4+ T-cell count below 350 cells/µL or the presence of an AIDS-defining illness at diagnosis [1], remains a persistent global barrier to optimal clinical outcomes. Indeed, individuals diagnosed at advanced stages face significantly higher risks for morbidity [2,3], mortality [2,3], drug resistance [4,5], and treatment complications, imposing a substantial burden on healthcare systems [2,3]. However, the drivers of this advanced immune depletion at diagnosis are multifaceted and not fully resolved.

Known contributors include direct viral cytotoxicity, chronic immune activation, increased apoptosis happening in infected and non-infected cells, and microbial translocation to list a few, have been described [6]. In short, both host factors and viral genetic diversity have been demonstrated to influence HIV disease progression. Moreover, HIV-1 subtypes including circulating recombinant forms differ in transmission dynamics, replication capacity, and immunopathogenesis, indicating that subtype-specific effects can also contribute to heterogeneity in immune status at presentation [7,8]. Concurrently, clinical context may reflect delayed diagnosis and systemic gaps in access [9]. To illustrate, the specific clinical circumstance triggering diagnosis, such as hospitalization, is a strong indicator of disease progression [10,11]. While inpatient diagnosis is recognized as a marker of advanced disease, its independent contribution to baseline immunological and biochemical impairment, relative to and in conjunction with viral and demographic factors, has not been systematically quantified in large ART-naïve cohorts.

Therefore, to address this gap, we conducted a retrospective cross-sectional analysis of ART-naïve newly diagnosed people living with HIV (PLWH). We aimed to disentangle the relative contributions of viral characteristics (HIV-1 subtype and drug resistance), demographic characteristics, and crucially, the clinical presentation context (inpatient vs. outpatient) to baseline immune and biochemical status. We hypothesized that the clinical context at diagnosis would be a stronger independent predictor of immune depletion than viral genetic factors, highlighting a critical and modifiable point for public health intervention.

## 2. Methods

### 2.1. Ethics Consideration

This study was conducted in accordance with the ethical principles outlined in the Declaration of Helsinki and received formal approval from the Ethics Committee of the Chongqing Public Health Medical Center, Chongqing, China (Approval No.: 2025-038-02-KY; Date of Approval: 25 December 2025). Given its retrospective design, the requirement for written informed consent was waived by the institutional review board. In lieu of consent, the confidentiality and privacy of all patient data were rigorously maintained in strict compliance with institutional and national clinical data protection protocols.

### 2.2. Study Population, Inclusion, and Exclusion Criteria

This retrospective, cross-sectional study focused on newly diagnosed and ART-naïve adult patients (≥18 years of age) who received an initial HIV diagnosis at our institution between January 2020 and October 2025. The study population included individuals presenting to either outpatient or inpatient services during this period. Our hospital serves as an HIV voluntary counseling and testing (VCT) site, a diagnosistic confirmation center, and the municipal referral center for treatment initiation and therapeutic quality control. Consequently, the study population encompassed both walk-in patients and those formally referred from other healthcare facilities across the region. The diagnostic protocol began with the detection of HIV-specific antibodies using two distinct immunoassays. All seropositive samples subsequently underwent confirmatory testing via Western blot analysis to identify definitive HIV protein bands. Additionally, plasma HIV RNA viral load (VL) was quantified in peripheral blood using quantitative reverse transcription polymerase chain reaction. To ascertain a genuinely new diagnosis, each case was verified against the national HIV case reporting registry. Individuals with no prior record in this system were formally classified as newly diagnosed and ART-naïve for the purposes of this study.

The study population was refined through the application of stringent exclusion criteria. Individuals were excluded if they: did not receive confirmatory HIV testing at our institution; had a previously documented HIV-positive diagnosis; had initiated any form of ART prior to baseline assessment; were receiving concomitant medication at the time of the initial HIV-related blood draw; or had undergone solid organ or hematopoietic stem cell transplantation. Further exclusions applied to patients with missing data for any core variable listed in the “clinical data collection and categorization” section, except for HIV-1 genotypic drug resistance testing result. Patients with serologically confirmed co-infection with hepatitis B (HBV) or hepatitis C (HCV) were also excluded.

### 2.3. Clinical Data Collection and Categorization

Clinical data were abstracted from the hospital’s electronic medical record (EMR) system for all individuals defined as ART-naïve newly diagnosed PLWH. Specifically, the initial laboratory report confirming HIV positivity was retrieved for each ART-naïve patient. To establish pre-treatment baselines, we reviewed immunological and biochemical profiles from blood tests ordered by clinicians prior to the commencement of ART. The extracted dataset encompassed the following variables: a unique patient ID, age, confirmed Western blot result, HIV-1 VL, CD4+ and CD8+ T-cell counts, the corresponding CD4+/CD8+ ratio, serum albumin (ALB) level, HIV-1 subtype, results of genotypic drug resistance testing, admission type (inpatient/outpatient), and hepatic transaminase levels, aspartate aminotransferase (AST) and alanine aminotransferase (ALT).

To refine the categorization, participants were stratified not only by admission type (inpatient or outpatient) but also by age. Following the precedent set in prior literature [12], two age-based cohorts were defined: an older cohort, comprising PLWH aged 50 years or older, and a younger cohort, comprising those under 50 years of age. Furthermore, the results of HIV-1 genotypic drug resistance testing were classified into three distinct categories: not tested, negative, and positive. The “not tested” category comprised cases in which genotypic resistance testing was not conducted due to financial constraints. The “negative” category referred to results indicating full susceptibility (sensitive), defined by the absence of resistance-associated mutations. Finally, the “positive” category indicated the detection of one or more such mutations, including those classified as potential resistance, low-level resistance, intermediate-level resistance, or high-level resistance.

### 2.4. Statistical Analysis

The data were collected on Microsoft excel and analyzed with GraphPad Prism software (version 10.2.2, Boston, MA, USA). Continuous variables were assessed for distributional normality using the Shapiro–Wilk test. Since most immunological and biochemical parameters were non-normally distributed, they are presented as medians with interquartile ranges (IQR), whereas normally distributed variables are reported as means with standard deviation (SD). Categorical variables are summarized as counts and percentages. Comparisons between two groups were performed using the Mann–Whitney U test for non-normally distributed continuous variables and the independent samples t-test for normally distributed variables. Comparisons across more than two groups were conducted using the Kruskal–Wallis test or one-way analysis of variance (ANOVA), as appropriate. Categorical variables were compared using the Chi square test or Fisher’s exact test when expected cell counts were small. Bivariate linear regression analyses were first conducted to evaluate associations between clinical and virological variables (age group, sex, admission type, HIV-1 subtype, genotypic drug resistance, ALT, and AST) and immunological/biochemical outcomes (CD4+ T-cell count, CD4+/CD8+ ratio, and serum albumin level). Variables with *p* ≤ 0.25 in bivariate analyses, as well as clinically relevant covariates, were entered into multivariate linear regression models to identify independent predictors. Regression coefficients (β) with corresponding 95% confidence intervals (95% CI) were calculated. Model assumptions, including linearity, homoscedasticity, and absence of multicollinearity, were assessed using residual diagnostics and variance inflation factors. All statistical tests were two-sided, and a *p* value <0.05 was considered statistically significant.

## 3. Results

### 3.1. Baseline Characteristics of the Study Population

**Figure 1:**
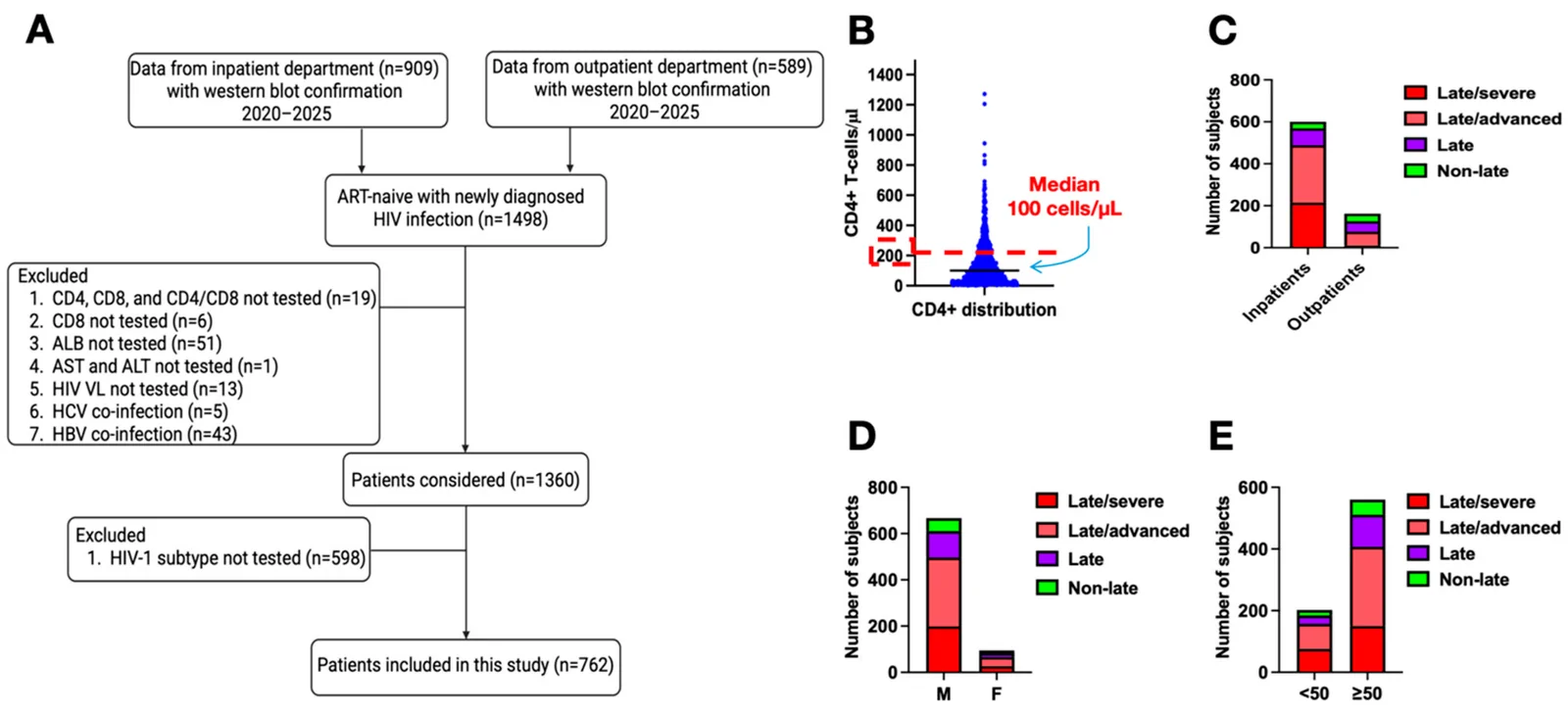
Study population and landscape of late HIV presentation among ART-naïve newly diagnosed PLWH. Panel (**A**) presents the study flow diagram. Panel (**B**) shows the distribution of CD4+ T-cell counts at diagnosis. Panels (**C**–**E**) illustrate late presentation categories stratified by admission type, sex, and age, respectively. M: male; F: female. Late/severe refers to patients with CD4+ T-cell counts below 50 cells/µL; Late/advanced refers to patients with CD4+ T-cell counts between 50 and below 200 cells/µL; Late refers to patients with CD4+ T-cell counts between 200 and below 350 cells/µL; Non-late refers to patients with CD4+ T-cell counts of 350 cells/µL or more.

A total of 762 ART-naïve newly diagnosed PLWH were included in this retrospective cross-sectional study (Figure 1 and Table 1). The median age was 59 (19), with 73.49% (560/762) of patients aged ≥50 years. Overall, 87.53% (667/762) were male. At diagnosis, 78.74% (600/762) of patients were managed as inpatients and 21.26% (162/762) as outpatients.

The median CD4+ T-cell count was 100 (167) cells/µL, median CD8+ T-cell count was 487.5 (494) cells/µL, and the median CD4+/CD8+ ratio was 0.2 (0.25). Mean serum ALB concentration was 34.8 (8.7) g/L. Median ALT and AST levels were 22 (23) IU/L and 28 (22) IU/L, respectively.

HIV-1 subtyping was successful in all of patients. The predominant strain was CRF07_BC (57.35%, 437/762), followed by CRF01_AE (16.14%, 123/762), CRF08_BC (15.88%, 121/762), subtype C (4.46%, 34/762), and others [1.57% (12/762) from CRF55_01B, 1.44% (11/762) from subtype B, 1.44% (11/762) from URF B/C, 0.66% (5/762) from subtype A, 0.66% (5/762) from CRF85_BC, 0.26% (2/762) from CRF34_01B, and 0.13% (1/762) from CRF52_01B]. Genotypic drug resistance testing was available for 98.95% (754/762) patients, of whom 22.15% (167/754) harbored at least one resistance-associated mutation.

### 3.2. Immunological Parameters According to HIV-1 Subtype

Significant differences in immunological markers were observed across HIV-1 subtypes. Patients with CRF01_AE had the lowest median CD4+ T-cell counts at diagnosis [54 (146) cells/µL] compared with CRF07_BC [113 (164) cells/µL], CRF08_BC [90 (175.5) cells/µL], subtype C [106.5 (118) cells/µL] and others [68 (213) cells/µL] (*p* = 0.0004). Notably, most of ART-naïve newly diagnosed PLWH were late presenters and the proportion of patients presenting with advanced immunosuppression (CD4+ T-cell < 200 cells/µL) was highest among those with subtype C (82.35%, 28/34). CRF01_AE (79.67%, 98/123) was following at the second position, then came CRF08_BC (72.73%, 88/121), others (72.34, 34/47), and CRF07_BC (72.31%, 316/437). Median CD8+ T-cell counts did not differ significantly by HIV-1 subtype (*p* = 0.3418), although CRF07_BC seem to have higher values (Table 1). The CD4+/CD8+ ratio was significantly lower in patients with CRF01_AE infection [0.15 (0.2)] compared with other subtypes (*p* = 0.0005). The details are provided in Table 2.

### 3.3. Biochemical Parameters by HIV-1 Subtype

Mean serum ALB levels did not differ significantly across HIV-1 subtypes (*p* = 0.1837). However, patients infected with CRF08_BC and CRF01_AE exhibited the lowest mean ALB concentrations [33.87 (5.76) g/L and 34.46 (6.42), respectively] among the subtypes analyzed. Elevated AST levels were more frequently observed in patients infected with CRF01_AE and other recombinant forms, who demonstrated relatively higher median AST values compared with other subtypes [32 (24) and 30 (24) vs. 27.5 (23), 27 (22.5), and 28 (20) IU/L for subtype C, CRF08_BC, and CRF07_BC, respectively; *p* = 0.5021, Table 2]. In addition, patients infected with subtype C showed relatively higher median ALT levels; however, ALT values did not differ significantly across HIV-1 subtypes (*p* = 0.7117).

### 3.4. Analysis by Genotypic Drug Resistance

Among ART-naïve newly diagnosed PLWH, individuals with detectable genotypic drug resistance mutations exhibited relatively lower median CD4+ T-cell counts and CD4+/CD8+ ratios compared to those without resistance [87 (185) vs. 101 (161) cells/µL and 0.18 (0.24) vs. 0.21 (0.25), respectively]; however, these differences did not reach statistical significance (*p* > 0.05, Table 3). In contrast, median CD8+ T-cell counts, mean serum ALB levels, and median AST and ALT values were comparable between patients with and without genotypic drug resistance, with no statistically significant differences observed.

### 3.5. Comparison by Admission Type

Inpatients presented with significantly more advanced immunological impairment than outpatients. Indeed, compared to outpatients, median CD4+ T-cell counts [76 (127.5) vs. 214 (207) cells/µL, *p* < 0.0001], CD8+ T-cell counts [421.5 (446.5) vs. 734 (614) cells/µL, *p* < 0.0001], and CD4/CD8 ratio [0.17 (0.22) vs. 0.28 (0.25), *p* < 0.0001] were significantly lower among inpatients. In the same line, mean serum ALB levels were significantly lower in inpatients compared with outpatients [33.13 (5.41) vs. 42.25 (5.75), *p* < 0.0001]. Inpatients also demonstrated higher median AST (*p* < 0.0001), but comparable median ALT levels (*p* = 0.3203). The HIV-1 subtype distribution was comparable between inpatients and outpatients, with CRF07_BC representing more than 50% of cases in each group. Notably, CRF07_BC was more frequently observed in outpatients than in inpatients (Table 4).

### 3.6. Comparison by Age Group

Interestingly, ART-naïve newly diagnosed PLWH aged ≥50 years had significantly higher median CD4+ T-cell counts at diagnosis compared with those aged <50 years [105.5 (165.5) vs. 74 (144) cells/µL, *p* = 0.0039]. CD8+ T-cell counts did not differ significantly between age groups, but the CD4+/CD8+ ratio was significantly higher among older patients too [0.21 (0.25) vs. 0.19 (0.22), *p* = 0.0173]. Mean serum ALB levels were lower in patients aged ≥50 years [34.39 (5.77) g/L, *p* < 0.0001]. ALT levels were significantly higher in the <50-year group [25 (29) vs. 21 (21) IU/L, *p* = 0.0386), while AST levels showed no significant age-related difference (Table 5).

### 3.7. Multivariate Linear Analysis

Bivariate and multivariate analyses demonstrated that admission type was the only factor independently associated with CD4+ T-cell count and serum ALB level. Notably inpatient admission was independently associated with lower CD4+ T-cell counts (β = −36.91, *p* < 0.0001) and reduced serum ALB levels (β = −7.358, *p* < 0.0001). Inpatient admission was also associated with lower CD4+/CD8+ ratios in bivariate analysis, but this association did not persist in multivariate analysis. Importantly, age, sex, HIV-1 subtype, genotypic resistance, ALT, and AST were not associated with CD4+ T-cell counts or CD4+/CD8 ratios in ART-naïve newly diagnosed PLWH. However, older age (≥50 years) and elevated AST levels were independently associated with lower serum ALB concentrations (Table 6).

## 4. Discussion

This study sought to disentangle the complex interplay of factors shaping the immunological and biochemical landscape at the time of HIV diagnosis. By simultaneously analyzing viral factors (including HIV-1 subtype and transmitted drug resistance), patient demographics (age < 50 vs. ≥50 years old), and crucially the clinical presentation context (inpatient vs. outpatient), we aimed to identify the strongest independent predictors of baseline disease severity.

Overall, our study revealed a striking burden of advanced immunosuppression at presentation, with nearly four in five patients classified as late presenters and a median CD4+ T-cell count of only 100 cells/µL (Figure 1). These findings underscore persistent gaps in early detection despite the availability of effective testing and treatment strategies. Similar results were subsequently documented in China, first in Wuhan by Tang et al., [9] and then in Xi’an by Jin et al., [13]. Furthermore, the most consistent signal emerging from our analysis is the strong association between inpatient admission and both immunological and nutritional compromise. As such, inpatients demonstrated substantially lower CD4+ T-cell counts, CD4+/CD8+ ratios, and serum ALB levels compared with outpatients, and inpatient status remained the only independent predictor of CD4+ T-cell decline and hypoalbuminemia (ALB levels < 35 g/L, Table 4) in multivariate models (Table 6). From a public health perspective, the substantial proportion of diagnoses made during hospitalization highlights missed opportunities for earlier detection through community-based testing initiatives, particularly in communities with a high concentration of older adults. Hospital admission should therefore be viewed not only as a clinical event but also as a sentinel indicator of failures in upstream screening systems.

Importantly, our findings revealed that clinical presentation at diagnosis may outweigh viral characteristics in determining host immunological status. Indeed, we observed significant subtype-associated differences in immunological markers, with CRF01_AE infection linked to the lowest CD4+ T-cell counts and CD4+/CD8+ ratios at diagnosis. This aligns with prior reports suggesting that CRF01_AE may be associated with faster CD4+ T-cell decline and more aggressive disease progression compared with other circulating recombinant forms [14,15]. Although subtype was not an independent predictor in multivariate analysis, the consistent unadjusted differences reported in Table 2 suggest that viral genetic background may influence host immune dynamics, potentially through differences in viral tropism, replication capacity, or immune activation pathways as indicated previously [16]. The high prevalence of CRF07_BC and CRF08_BC reflects their predominance in the regional epidemic; these subtypes originated in Yunnan province before expanding across Asia [17,18]. This predominance and their persistent evolution could significantly impact disease progression models, immune recovery post-ART, and vaccine design. Therefore, future longitudinal studies are essential to determine whether the baseline differences identified here lead to divergent long-term clinical outcomes. Besides HIV subtypes, we noted that approximately one-fifth of ART-naïve individuals harbored resistance-associated mutations (Table 3), a prevalence that raises concern for transmitted drug resistance. However, resistance status was not associated with baseline immunological or biochemical parameters. This suggests that transmitted resistance does not necessarily confer a detectable early immunological disadvantage at diagnosis. Nevertheless, its public health significance remains substantial, as it may compromise first-line regimen efficacy and increase long-term healthcare burden.

Interestingly, older individuals (≥50 years) presented with higher CD4+ T-cell counts and CD4+/CD8+ ratios than younger patients, despite exhibiting lower ALB levels (Table 5). This paradox may result from a selection bias as older adults tend to present with significantly lower CD4+ T-cell counts at diagnosis [19]. In addition, this paradox may also reflect differences in healthcare-seeking behavior, comorbidity-driven testing, or survivor bias. Indeed, older adults may undergo more frequent medical evaluations, leading to earlier incidental HIV detection, whereas younger individuals may present only after symptomatic progression. Future investigations in our particular settings will help to explain our observation. At the same time, reduced ALB levels in older patients likely capture age-related frailty, chronic inflammation, or comorbid conditions independent of HIV-mediated immunosuppression [20,21,22,23]. The preceding findings highlight the need to consider aging and HIV as intersecting biological processes. Nutritional and metabolic vulnerability in older PLWH may not parallel immunological status and as such should be addressed through integrated care models.

While the present study provides valuable insights into immune and biochemical phenotypes in ART-naïve newly diagnosed PLWH, several limitations merit consideration. First, its retrospective cross-sectional design does not permit causal inference or the assessment of disease progression and treatment outcomes. Second, the reliance on single-timepoint laboratory measurements limits our ability to characterize the dynamic evolution of immune responses. Third, despite statistical adjustments, unmeasured or residual confounding (uncontrolled period of infection, transmission route, viral load, etc.), including by comorbidities, socioeconomic factors, or undiagnosed opportunistic infections, may influence the reported associations. To elaborate further, nutritional status, along with socioeconomic and educational background, is not routinely documented in newly diagnosed HIV patients, which precluded us from assessing these potential confounding factors. Moreover, data on AIDS-defining illnesses, opportunistic infections, and comorbidities were available only for a subset of inpatients, and were largely unattainable in the outpatient setting (see Supplementary Table S1), severely constraining our ability to rigorously adjust for these variables. Nevertheless, we view this study as a valuable opportunity to underscore the need for improved data collection practices in newly diagnosed patients, thereby facilitating higher-quality analyses in future research. Finally, the cohort reflects a specific regional epidemic and care landscape; therefore, findings may not be fully generalizable to populations with differing HIV-1 subtype distributions, host genetics, or healthcare access patterns.

## 5. Conclusions

Our findings reveal that the overwhelming prevalence of severe immunodeficiency at HIV diagnosis is less a consequence of viral genetic variation than a reflection of advanced clinical disease. The emergence of inpatient admission as the paramount independent predictor of both CD4+ T-cell collapse and hypoalbuminemia suggests that presentation severity captures a state of broader host vulnerability, extending beyond virological factors. While subtle subtype-related differences were observable, their disappearance after multivariate adjustment underscores the primacy of the host clinical context. These insights reframe late diagnosis from a seeming virological inevitability to a structural failure of healthcare systems. Thus, efforts to improve early detection, particularly in aging cohorts, are likely to confer greater immunological benefit than approaches focused on viral characteristics. Future risk stratification models that incorporate immunological and metabolic markers at presentation could optimize the identification of patients requiring the most intensive early intervention.

## Figures and Tables

**Table 1:** Baseline demographic, clinical, immunovirological, and biochemical characteristics.

Variables	Total (*n* = 762)
Sex	
Male	667
Female	95
Age	
<50	202
≥50	560
Admission	
Inpatient	600
Outpatient	162
HIV-1 subtype	
C	34
CRF01_AE	123
CRF07_BC	437
CRF08_BC	121
Others	47
Geno-DR	
Not tested	8
Positive	167
Negative	587
CD4+ T-cell count	100
[median (IQR)]	(167)
CD8+ T-cell count	487.5
[median (IQR)]	(494)
CD4+/CD8+ ratio	0.20
[median (IQR)]	(0.25)
ALB	35.07
[Mean (SD)]	(6.63)
AST	28
[median (IQR)]	(22)
ALT	22
[median (IQR)]	(23)
HIV VL	398,500
[median (IQR)]	(1,192,000)

**Table 2:** Immunovirological and biochemical parameters according to HIV-1 subtype.

Variables	Subtype C (*n* = 34)	CRF01_AE (*n* = 123)	CRF08_BC (*n* = 121)	CRF07_BC (*n* = 437)	Others (*n* = 47)	*p*
CD4+ T-cell [median (IQR)]	106.5 (118)	54 (146)	90 (175.5)	113 (164)	68 (213)	0.0004
CD8+ T-cell [median (IQR)]	440 (606)	407 (533)	490 (495.5)	504 (472)	417 (571)	0.3418
CD4+/CD8+ [median (IQR)]	0.23 (0.22)	0.15 (0.2)	0.21 (0.22)	0.21 (0.27)	0.20 (0.24)	0.0005
HIV VL [median (IQR)]	402,000 (1,746,000)	432,000 (1,149,000)	337,000 (909,050)	410,000 (1,326,500)	451,000 (819,300)	0.7934
ALB [Mean (SD)]	34.92 (5.26)	34.46 (6.42)	33.87 (5.76)	35.42 (6.8)	36.65 (8.08)	0.1837
ALT [median (IQR)]	27.5 (27)	22 (28)	21 (19.5)	23 (22)	20 (23)	0.7117
AST [median (IQR)]	27.5 (23)	30 (24)	27 (22.5)	28 (20)	32 (24)	0.5021

**Table 3:** Comparison of immunological and biochemical parameters by genotypic drug resistance status.

Variables	Geno-DR Negative (*n* = 587)	Geno-DR Positive (*n* = 167)	*p*
CD4+ T-cell [median (IQR)]	101 (161)	87 (185)	0.1254
CD8+ T-cell [median (IQR)]	493 (497)	431 (504)	0.2078
CD4+/CD8+ [median (IQR)]	0.21 (0.25)	0.18 (0.24)	0.2574
HIV VL [median (IQR)]	425,000 (1,233,000)	336,000 (958,000)	0.4702
ALB [Mean (SD)]	35.18 (6.75)	34.71 (6.34)	0.4169
ALT [median (IQR)]	22 (23)	22 (23)	0.7057
AST [median (IQR)]	28 (22)	28 (22)	0.6180

**Table 4:** Comparison of parameters by admission type.

Variables	Inpatients (*n* = 600)	Outpatients (*n* = 162)	*p*
CD4+ T-cell [median (IQR)]	76 (127.5)	214 (207)	<0.0001
CD8+ T-cell [median (IQR)]	421.5 (446.5)	734 (614)	<0.0001
CD4+/CD8+ [median (IQR)]	0.17 (0.22)	0.28 (0.25)	<0.0001
HIV VL [median (IQR)]	499,000 (1,423,500)	144,500 (425,000)	<0.0001
ALB [Mean (SD)]	33.13 (5.41)	42.25 (5.75)	<0.0001
ALT [median (IQR)]	23 (22.5)	22 (21)	0.3203
AST [median (IQR)]	30 (24)	24 (13)	<0.0001
Subtype C	31 (5.17%)	3 (1.85%)	0.0696
CRF01_AE	103 (17.17%)	20 (12.35%)	0.1393
CRF07_BC	332 (53.33%)	105 (64.81%)	0.0091
CRF08_BC	103 (17.17%)	18 (11.11%)	0.0613
Others	31 (5.17%)	16 (9.88%)	0.0272

**Table 5:** Comparison of parameters by age group.

Variables	<50 Years (*n* = 202)	≥50 Years (*n* = 560)	*p*
CD4+ T-cell [median (IQR)]	74 (144)	105.5 (165.5)	0.0039
CD8+ T-cell [median (IQR)]	459.5 (476)	498 (507)	0.0928
CD4+/CD8+ [median (IQR)]	0.19 (0.22)	0.21 (0.25)	0.0173
HIV VL [median (IQR)]	418,000 (1,242,300)	395,000 (1,171,000)	0.9137
ALB [Mean (SD)]	36.97 (8.31)	34.39 (5.77)	<0.0001
ALT [median (IQR)]	25 (29)	21 (21)	0.0386
AST [median (IQR)]	28.5 (28)	28 (20)	0.0778

**Table 6:** Predictors of CD4+ T-cell counts, CD4+CD8+ ratios, and ALB levels.

Bivariate Analysis												
Variables	CD4 +T-cell				CD4/CD8 Ratio				ALB			
	β	95% CI		*p*	Estimate	95% CI		*p*	Estimate	95% CI		*p*
		Lower	Upper			Lower	Upper			Lower	Upper	
Sex												
F	1				1				1			
M	−13.10	−45.87	19.68	0.4330	−0.01	−0.06568	0.04521	0.7173	−1.31	−2.740	0.1118	0.0708
Age												
<50 years	1				1				1			
≥50 years	11.55	−12.97	36.08	0.3554	0.0217	−0.0197	0.0632	0.3040	−2.58	−3.637	−1.530	<0.0001
Admission												
Outpatient	1				1				1			
Inpatient	−127.7	−152.6	−102.9	<0.0001	−0.0727	−0.1172	−0.0283	0.0014	−9.121	−10.07	−8.168	<0.0001
HIV-1												
Others	1				1				1			
Subtype C	10.93	−56.34	78.20	0.7498	0.035	−0.078	0.149	0.5425	−1.730	−4.652	1.193	0.2456
CRF01_AE	−26.72	−77.96	24.52	0.3063	−0.018	−0.105	0.067	0.6720	−2.193	−4.419	0.033	0.0534
CRF07_BC	1.36	−44.51	47.23	0.9536	0.043	−0.035	0.120	0.2816	1.015	−3.230	0.754	0.2229
CRF08_BC	−11.58	−62.94	39.77	0.6581	0.014	−0.073	0.100	0.7563	−2.782	−5.013	−0.551	0.0146
Geno-DR												
Not tested	1				1				1			
Negative	22.18	−84.25	128.6	0.6825	0.107	−0.073	0.286	0.2444	0.7824	−3.856	5.421	0.7407
Positive	11.43	−96.79	119.6	0.8358	0.095	−0.088	0.278	0.3077	0.3080	−4.409	5.025	0.8980
ALB	9.01	7.51	10.51	<0.0001	0.006	0.003	0.008	<0.0001	-	-	-	-
ALT	0.081	−0.10	0.263	0.3867	0.0001	−0.0002	0.0004	0.5103	−0.0012	−0.0091	0.0068	0.7726
AST	−0.30	−0.59	−0.014	0.0400	−7.6 × 10^−5^	−0.0005	0.0004	0.7614	−0.0308	−0.0433	−0.0184	<0.0001
HIV VL	−1.5 × 10^−7^	−1.5 × 10^−6^	1.2 × 10^−6^	0.8285	−10^−9^	−3 × 10 ^−9^	1.2 × 10^−9^	0.3819	−1.6 × 10^−8^	−7.5 × 10^−8^	4.3 × 10^−8^	0.5950
CD8+	0.12	0.106	0.1367	<0.0001	−6.3 × 10^−5^	−9.3 × 10^−5^	−3.4 × 10^−5^	<0.0001	0.00244	0.00169	0.00319	<0.0001
CD4/CD8 ratio	329.9	294.9	364.8	<0.0001	-	-	-	-	3.697	1.881	5.513	<0.0001
CD4+	-	-	-	-	0.0009	0.0008	0.001	<0.0001	0.01711	0.01426	0.01997	<0.0001
**Multivariate analysis**												
**Variables**	**CD4 +T-cell**				**CD4/CD8 ratio**				ALB			
	**β**	**95% CI**		***p***	**Estimate**	**95% CI**		***p***	**Estimate**	**95% CI**		***p***
		**Lower**	**Upper**			**Lower**	**Upper**			**Lower**	**Upper**	
Sex	-	-	-	-	-	-	-	-				
F									1			
M									0.6588	−0.4883	1.806	0.2599
Age	-	-	-	-	-	-	-	-				
<50 years									1			
≥50 years									−1.958	−2.827	−1.088	<0.0001
Admission												
Outpatient	1				1				1			
Inpatient	−36.91	−54.98	−18.83	<0.0001	0.034	−0.003	0.071	0.0697	−7.358	−8.370	−6.345	<0.0001
HIV-1	-	-	-	-	-	-	-	-				
Others									1			
Subtype C									0.8424	−1.504	3.189	0.4811
CRF01_AE									−0.1141	−1.892	1.664	0.8998
CRF07_BC									−0.1484	−1.738	1.442	0.8546
CRF08_BC									−0.7913	−2.581	0.9981	0.3856
Geno-DR	-	-	-	-					-	-	-	-
Not tested					1							
Negative					0.05961	−0.06089	0.1801	0.3318				
Positive					0.05984	−0.06263	0.1823	0.3378				
ALB	2.885	1.745	4.026	<0.0001	−0.00153	−0.00385	0.00077	0.1912	-	-	-	-
AST	−0.056	−0.222	0.109	0.5044	-	-	-	-	−0.02017	−0.03027	−0.01006	<0.0001
CD8+	0.132	0.1220	0.143	<0.0001	−0.00023	−0.00025	−0.00021	<0.0001	0.00016	−0.00071	0.0010	0.7204
CD4/CD8 ratio	360.3	335.9	384.7	<0.0001	-	-	-	-	−1.085	−3.258	1.089	0.3275
CD4+	-	-	-	-	0.00146	0.00136	0.00156	<0.0001	0.01083	0.00653	0.01513	<0.0001

95% confidence interval: 95% CI.

## Data Availability

Upon request, and subject to review, the corresponding author will provide the data that support the findings of this study.

## Supplementary Materials

The following supporting information can be downloaded at: https://www.mdpi.com/article/10.3390/v18090986/s1, Table S1: Overview of documented comorbid conditions and clinical presentations at diagnosis in ART-naïve, newly identified PLWH, stratified by inpatient versus outpatient care settings.
