# Supplementary material for: Late Clinical Presentation Outweighs Viral Characteristics in Determining Immune Depletion at HIV Diagnosis: A Five-Year Retrospective Study
Source: Viruses. 2026 Sep 8;18(9):986. doi: 10.3390/v18090986 (PMC13611979; doi:10.3390/v18090986)
Supplement: Supplementary file 1 [file viruses-18-00986-s001.zip › viruses-4518289-supplementary.pdf]

Supplementary Table S1. Overview of documented comorbid conditions and clinical presentations at diagnosis in ART-naïve, newly identified PLWH, stratified by inpatient versus outpatient care settings.

Conditions at HIV diagnosis	Inpatients (n)	Outpatients (n)
Acute cerebral infarction	2	-
Acute cerebral ischemia	2	-
Acute tonsillitis	2	-
AIDS (with multiple coinfections)	285	1
Anal fissure	2	-
Atherosclerotic gangrene of the right lower extremity	1	-
Bacterial gastroenteritis	2	-
Bacterial pneumonia	7	-
Bronchitis	2	-
Cervical disc herniation	1	-
Cervical lymphadenopathy (unknown etiology)	5	-
Cervical lymphadenopathy (tuberculosis with abscess)	1	-
Cervical spondylosis	2	-
Chronic gastritis with erosions	3	-
Chronic kidney disease stage 3	1	-
COPD	9	-
Diarrhea (etiology unknown)	5	-
Diffuse large B-cell lymphoma	4	-
Dizziness (etiology unknown)	9	-
Fatigue (unknown etiology)	2	-
Femoral fracture	2	-
Fever	8	-
Finger cellulitis	1	-
Gastrointestinal bleeding	2	-
Genital ulceration	1	-
Hemoptysis (unknown etiology)	1	-
Herpes simplex virus type 2 infection	1	-
Herpes zoster infection	4	-
Hypopharyngeal squamous cell carcinoma	1	-
Impaired consciousness (unknown etiology)	1	-
Inguinal lymph node tuberculosis	1	-
Intra-abdominal infection	1	-
Intracranial infection (unknown etiology)	7	-
Left hand abscess	1	-
Left inguinal hernia	1	-
Lymphoma	1	-
Meningitis (*Cryptococcus neoformans*)	4	-
Tuberculous meningitis	1	-
Multiple cerebral ischemia	1	-
Multiple colonic polyps	2	-
Neurosyphilis	1	-
Non-Hodgkin lymphoma	1	-
Numbness of the limbs (unknown etiology)	1	-
Oral candidiasis	4	-
Perianal abscess	6	-

Pulmonary infection (unknown etiology)	78	-
Pulmonary nodule (unknown etiology)	13	-
Pulmonary tuberculosis	29	-
Rectal cancer	1	-
Redness and swelling of the foreskin (unknown etiology)	1	-
Acute renal failure	3	-
Chronic renal failure	1	-
Renal insufficiency	2	-
Retinitis	1	-
Right ureteral stone with infection	1	-
Right-sided hydrocele of the testis	1	-
Sepsis	1	-
Septicemia	2	-
Severe PCP	8	-
Small-cell lung cancer, stage IV	1	-
Swollen lymph nodes (tuberculous lymphadenitis)	2	-
Swollen lymph nodes (unknown etiology)	1	-
Symptomatic epilepsy; sequelae of cerebral hemorrhage	1	-
Thrombocytopenia	7	-
Thyroid cyst	1	-
Toxoplasmic encephalitis	1	-
Tuberculous pleurisy	2	-
Type 2 diabetes	4	-
Urinary tract infection	3	-
Not reported	33	161
**Total**	**600**	**162**

Abbreviations: AIDS, Acquired immunodeficiency syndrome; COPD, chronic obstructive pulmonary disease; PCP, Pneumocystis pneumonia. A hyphen (–) indicates no reported coinfections, opportunistic infections, or comorbidities.
